# Feeding systems influence the rumen resistome in yaks by changing the microbiome

**DOI:** 10.3389/fmicb.2025.1505938

**Published:** 2025-03-19

**Authors:** Shuli Yang, Jialuo Chen, Jieyi Zheng, Huaming Mao, Feilong Deng, Dongwang Wu, Jianmin Chai

**Affiliations:** ^1^Guangdong Provincial Key Laboratory of Animal Molecular Design and Precise Breeding, School of Animal Science and Technology, Foshan University, Foshan, China; ^2^Key Laboratory of Animal Nutrition and Feed Science of Yunnan Province, Yunnan Agricultural University, Kunming, China

**Keywords:** antibiotics, rumen, metagenomics, microbiome, ruminants, resistome, yaks

## Abstract

The rumen microbiome serves as a reservoir of antibiotic-resistance genes (ARGs) with significant implications for public health. This study aimed to investigate the effects of different feeding systems on the rumen resistome in yaks. Yaks that grazed naturally on pasture were used as controls, while the experimental yaks were housed in a high-density pen environment and fed a specially designed diet to optimally meet their nutritional requirements, with increased interactions with farm workers. Metagenomic analysis was performed to assess changes in the rumen microbiome and resistome. Dietary factors influencing changes in the rumen microbiome and resistome were identified. A greater variety of microbiomes associated with carbohydrate digestion was found in yaks under a house-feeding system, such as *Stomatobaculum longum* and *Succiniclasticum ruminis.* Although grazing yaks exhibited various dominant antibiotic resistance genes (ARGs) at the class level, house-fed yaks were mainly enriched with tetracycline-resistant genes. A random forest model identified specific ARG signatures for each group, such as Sent_cmlA and Sliv_cmlR (Phenicol) and vanHD (Glycopeptide) prevalent in grazing yaks, while tet44, tetW, tetW/N/W, and tet40 were abundant in house-fed yaks. ARG interactions varied by feeding system, with signature ARGs in each group showing distinct correlations. Nevertheless, strong correlations among ARGs existed regardless of the treatments, such as the positive correlation between tetW and tetW/N/W in both groups. The rumen microbiome was strongly associated with the resistome, especially regarding abundant microbiomes and ARGs. Proteobacteria carrying ARGs were observed in grazing yaks, while Firmicutes served as hosts for ARGs in yaks under a housed feeding system. The specific bacteria contributing to the distinct ARGs in each group were identified. For instance, members of Firmicutes (*Clostridium tepidiprofundi*) carried their ARG signatures, such as tet44. These findings emphasized that diet, along with environmental factors and farmworker interactions, contributed to changes in the rumen resistome of yaks. This study is the first to discuss how multiple factors within a feeding regime influence the gut resistome, highlighting the drawbacks of intensive feedings with respect to the gut resistome.

## Graphical abstract

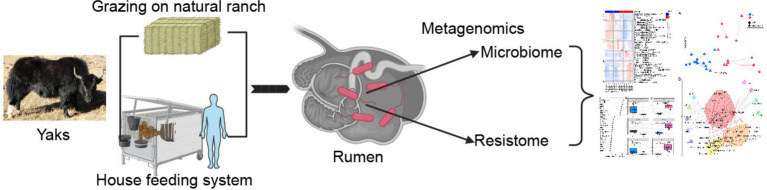

## Background

Yak (*Bos grunniens*) is a unique and important ruminant found in high-altitude regions. It has adapted to extreme conditions, including harsh winters, low oxygen, and high ultraviolet radiation (Ge et al., 2013; Qiu et al., 2015). Traditionally, yaks graze annually on natural pastures without any dietary supplementation. Recently, pasture-based grazing systems with concentrate diet supplements or complete house feeding systems have been introduced for yaks. This alteration in the feeding system has improved growth performance, rumen fermentation, and gut microbiota composition (Dai et al., 2022; Dai R. et al., 2023; Liu et al., 2023; Yang et al., 2024). A house-feeding system that has been widely applied to other livestock provides a specially formulated diet to animals living in restricted high-density pens, resulting in increased productivity (Moorby and Fraser, 2021).

Modifying the diet from natural pasture feed to a formulated diet precisely meets the animals’ nutrient requirements and subsequently alters the rumen microbiota. These microbial communities ferment dietary plant components into volatile fatty acids (VFAs) and microbial proteins, which contribute to host production (Chai et al., 2021). One study found that fattening with a formulated diet altered rumen fermentation, microbial diversity, and meat quality in yaks (Hu C. et al., 2021). Another study observed that warm-grazing and cold-indoor feeding regimes affected the yak rumen microbiome and greenhouse gas emissions compared to traditionally grazing yaks (Zhang et al., 2022). Thus, alterations in the feeding system, particularly dietary factors, influence the rumen microbiome in yaks.

The spread of antibiotic resistance genes (ARG) is a major public health concern, with farms serving as key reservoirs. The use of antibiotics in the animal industry is approximately three times higher than in humans (Van Boeckel et al., 2019). In 2020, China used approximately 33,000 tons of antimicrobials in food animals, with tetracyclines, macrolides, and *β*-lactams being the most commonly used antibiotics (Zhao et al., 2023). High levels of antibiotics contribute to the emergence and persistence of ARGs and antibiotic-resistant microbiota (Ding et al., 2022), resulting in decreased efficacy of therapeutic antibiotics. Compared to traditional grazing systems with minimal antibiotic exposure, the housed feeding system used in the yak industry may exacerbate these negative effects due to high-density feeding, frequent contact with raisers, care by clinical veterinarians, and regular antibiotic use (Wang et al., 2021).

The widespread use of antibiotics in livestock can facilitate ARG transmission to humans through various pathways. Recent studies have confirmed that farm exposure leads to interconnectedness among microbiomes and ARGs in the nasal, oropharyngeal, and gut communities of farmers, cattle, and their environments (Ding et al., 2022; Mahmud et al., 2024). Moreover, potential microbiome transmission from animal products to the human gut has been reported (Carlino et al., 2024). Thus, foodborne pathogens acquiring ARGs through horizontal or vertical gene transfer can contribute to difficult-to-treat infectious diseases (Kim and Ahn, 2022). In ruminants, the rumen microbiome is recognized as a reservoir of ARGs (Auffret et al., 2017), which is affected by dietary changes and associated with ARG threats (Chai et al., 2024a). Given the relationship between rumen ARGs and microbiota (Amin et al., 2021), transitioning from a grazing system to a housed feeding system may also affect the rumen resistome (the complete set of antibiotic resistance genes). However, no related studies have been conducted.

We hypothesize that the house-feeding environment remodels the yak rumen resistome and enriches ARG selection. Therefore, in this study, we conducted rumen metagenomics to identify the impacts of a house-feeding system on the rumen resistome and microbiome in yaks compared to those in a traditional grazing regime. This study will elucidate how the new environment, workers, and diet in a house feeding system comprehensively affect the rumen resistome, thereby enhancing our understanding of ARG dissemination in the farming environment.

## Materials and methods

### Animals and sampling

The current study was conducted under the guidance of the Animal Ethics Committee at Yunnan Agricultural University (approval number: 202009006). The experiment adhered to the Animal Research: Reporting of *In Vivo* Experiments (ARRIVE) guidelines. The animal trial took place at Tiancheng Lun Zhu Agricultural Products Development Co., Ltd., located at an altitude of approximately 3,600 m, to the north of Shangri-La County, Yunnan Province, China.

A total of 14 healthy yaks (aged 3 to 4 years) were randomly assigned to two groups: (1) yaks that traditionally grazed all day on the natural pasture (Control group, C); (2) yaks that were raised under a house-feeding system (House Feeding group, H), which provided a total mixed ration diet. Specifically, yaks in the control group roamed freely on natural pastures with minimal artificial intervention, such as unrestricted forage access, no supplementation feeding, and no veterinary care In contrast, yaks in the house feeding group were kept in a pen with limited space, receiving a specially designed diet to meet the expected growth rate, along with care from clinical veterinarians. The animal trial lasted 60 days.

In the C group, the natural pasture provided forage that included *Potentilla fulgens*, *Blysmus sinocompressus*, *Poa pratensis*, *Carex tristachya*, *Spiranthes sinensis*, and *Euphorbia joking*. The diet for the H group consisted of whole silage corn, ground corn, soybean meal, rapeseed meal, corn gluten meal, vinasse, and a premix. The H group was fed twice daily. All animals involved in this study were healthy and did not receive any recorded therapeutic or prophylactic antibiotic treatments.

At the end of the trial, rumen content samples were collected from each yak using an oral stomach tube, following a previously established method (Yang et al., 2024). Briefly, at 8 am, a tube was inserted into the rumen through the yak’s mouth, and a vacuum sampler was used to pump rumen fluid. The first 30 mL of the rumen digesta sample was discarded to prevent saliva contamination. For each animal, 30 mL of rumen fluid was collected and divided into three portions, each placed in a 10-mL polypropylene tube. The collected rumen fluid was rapidly stored in liquid nitrogen and subsequently kept in a freezer at −80°C for metagenomic analysis.

### Shotgun metagenomic sequencing

Total genomic DNA from the rumen content samples was extracted using a DNeasy PowerSoil Kit (Qiagen, Hilden, Germany) according to the manufacturer’s instructions. The integrity of the DNA was assessed using 1% agarose gel electrophoresis. Negative controls were included during both the extraction and PCR processes. A Qubit 2.0 fluorimeter (Invitrogen, Carlsbad, California, USA) was employed to measure the DNA concentration of the samples. The sequencing library was constructed using a TruSeq DNA PCR-Free Library Preparation Kit (Illumina, San Diego, California, USA), which was efficient and provided comprehensive fragment coverage. Metagenomic sequencing was conducted on an Illumina HiSeq 4,000 platform, known for its stability and high genome coverage, at the Beijing Headquarters of Novogene Biotech Co., Ltd. (Beijing, China). All experimental procedures were conducted in a stringent, clean, and sterile Class II B2 biosafety chamber to minimize contamination. Metagenomic sequencing data have been deposited in the NCBI SRA database (PRJNA1026838).

Trimmomatic (version 0.33) was used to remove low-quality reads with quality scores below 20, short reads (< 40 bp), and “N” bases using the default settings. To eliminate host contamination, the reads were aligned with the yak genome (BosGru3.1) to filter out reads of host origin (Langmead and Salzberg, 2012). Next, the clean data were assembled for each sample using SOAPdenovo software (V2.04) with default settings (Luo et al., 2012). MetaGene was utilized to predict open reading frames (ORFs) (Noguchi et al., 2006). The ORFs with lengths≥ 100 bp were retrieved and translated into amino acid sequences using the NCBI translation table. A non-redundant gene catalog was constructed using CD-HIT with 95% identity and 90% coverage (Fu et al., 2012).

## Rumen microbiome and resistome classification

Regarding microbial taxonomic classification, DIAMOND software (V0.9.9) (Buchfink et al., 2015) was used to blast sequences against the nr database (Version: 2023-07-28) with the parameter settings blastp -e 1e-5. The taxonomic profiles of the rumen microbiome were analyzed to compare the effects of the two feeding systems. Beta diversity, based on Bray-Curtis distances, was calculated and visualized using PCoA plots. Microbial taxa with a relative abundance exceeding 0.1% in at least 50% of yaks in each group were identified to characterize the signature for each group.

To classify ARGs within the assembly, we used the Comprehensive Antibiotic Resistance Database (CARD) (McArthur et al., 2013). In order to achieve highly accurate identification of ARGs, ORFs within the assembly were selected using the “longest-orf” option and were subsequently annotated against the CARD database using DIAMOND (V0.9.9). The annotation output was converted into a GFF file and filtered for overlap using MGKIT, with a bitscore threshold of 60.0 and a minimum percentage identity of 75%. Annotations related to resistance conferred by mutations were eliminated to prevent the false identification of non-mutated genes in the results. HTSeq-count (v0.6) was employed to count the number of reads from each sample aligning with each CARD-annotated ORF. The parameters used included a minimum alignment quality of 8 and the intersection-nonempty overlap resolution mode. The read counts for each sample were scaled to the minimum sample size to prevent sample size bias.

### Statistics

Rumen microbiomes were compared at the domain, phylum, and genus levels using the Wilcoxon rank-sum test, as these data were unnormalized. The dissimilarity of the rumen microbiome and resistome was assessed using Analysis of Similarity (ANOSIM) via the “adonis2” function in the R “vegan” package. Metastats were used to classify the signature microbiome differentiating these two groups (White et al., 2009). A random forest model, an ensemble learning method for high-dimensional data classification, was conducted to identify ARG signatures. The RandomForest v.4.6–7 package in R was used to perform the random forest process. All ARGs were included as variables, with the “importance” and “proximity” parameters set to “True” and “ntree” set to 10.000. The variable importance plot was generated based on the importance scores (mean decrease accuracy) of features. In this study, the top 25 features were considered important predictors. For network analysis, Spearman’s correlation was used to calculate correlations between features using the “psych” package in R, and the network was visualized using Cytoscape (version 3.10.1). Only significant connections were included in the network (|r| > 0.7, *p* < 0.05). The Linear Discriminant Analysis Effect Size (LEfSe) algorithm (Segata et al., 2011) was used via the Galaxy online server^1^ to detect the microbial origin of ARGs differentiating C and H groups. The cutoff for significant differences in LEfSe analysis was set at LDA score > 2 and *p*-value <0.05.

## Results

### House feeding system significantly changed the composition of the rumen in yaks

**Figure 1:**
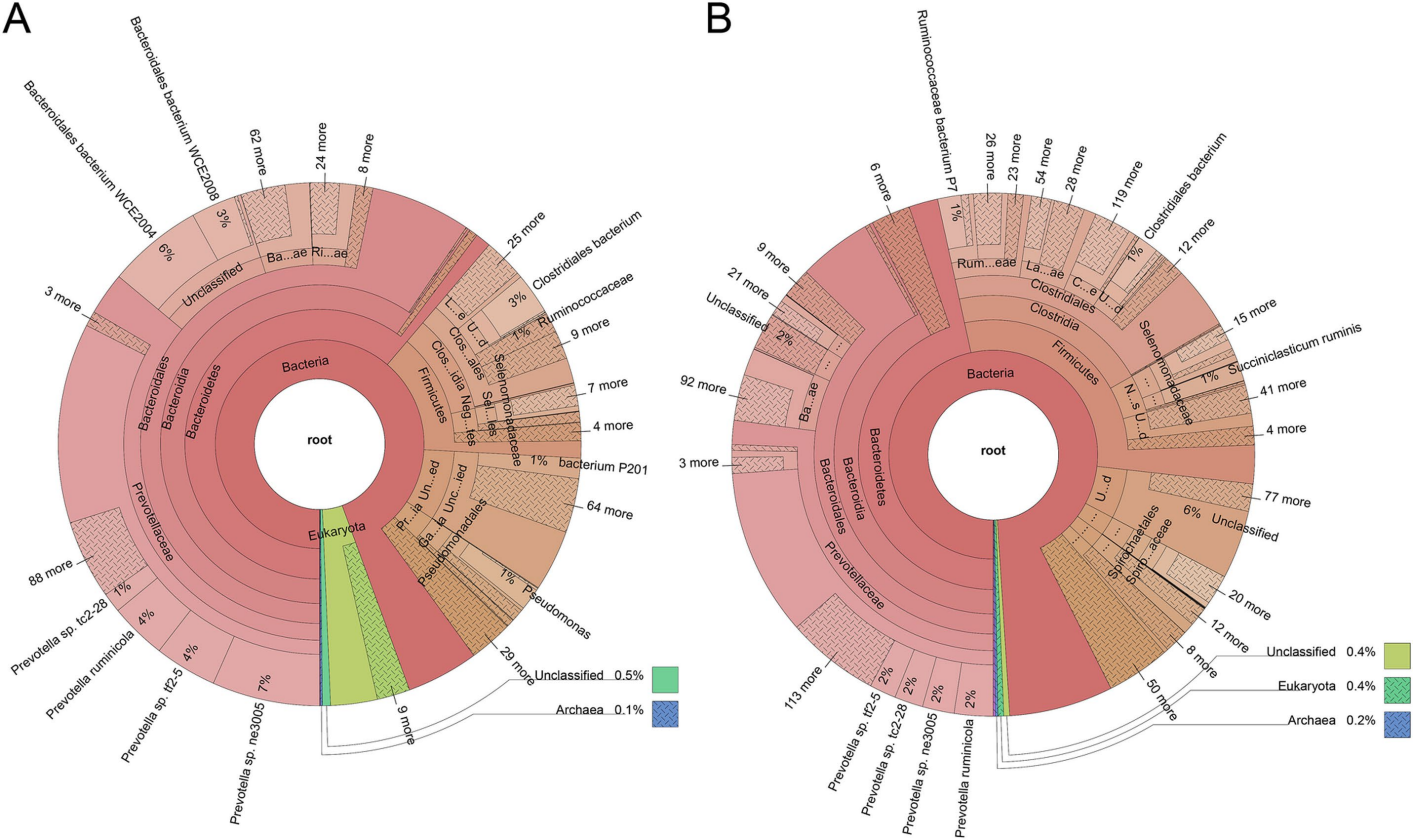
The house feeding system altered the rumen microbiome composition in yaks. The rumen metagenome from a grazing system **(A)** and a housed feeding system **(B)** were displayed using Krona. Taxonomy nodes are represented as nested sectors, arranged with the top level of the hierarchy in the center and progressing outward.

**Figure 2:**
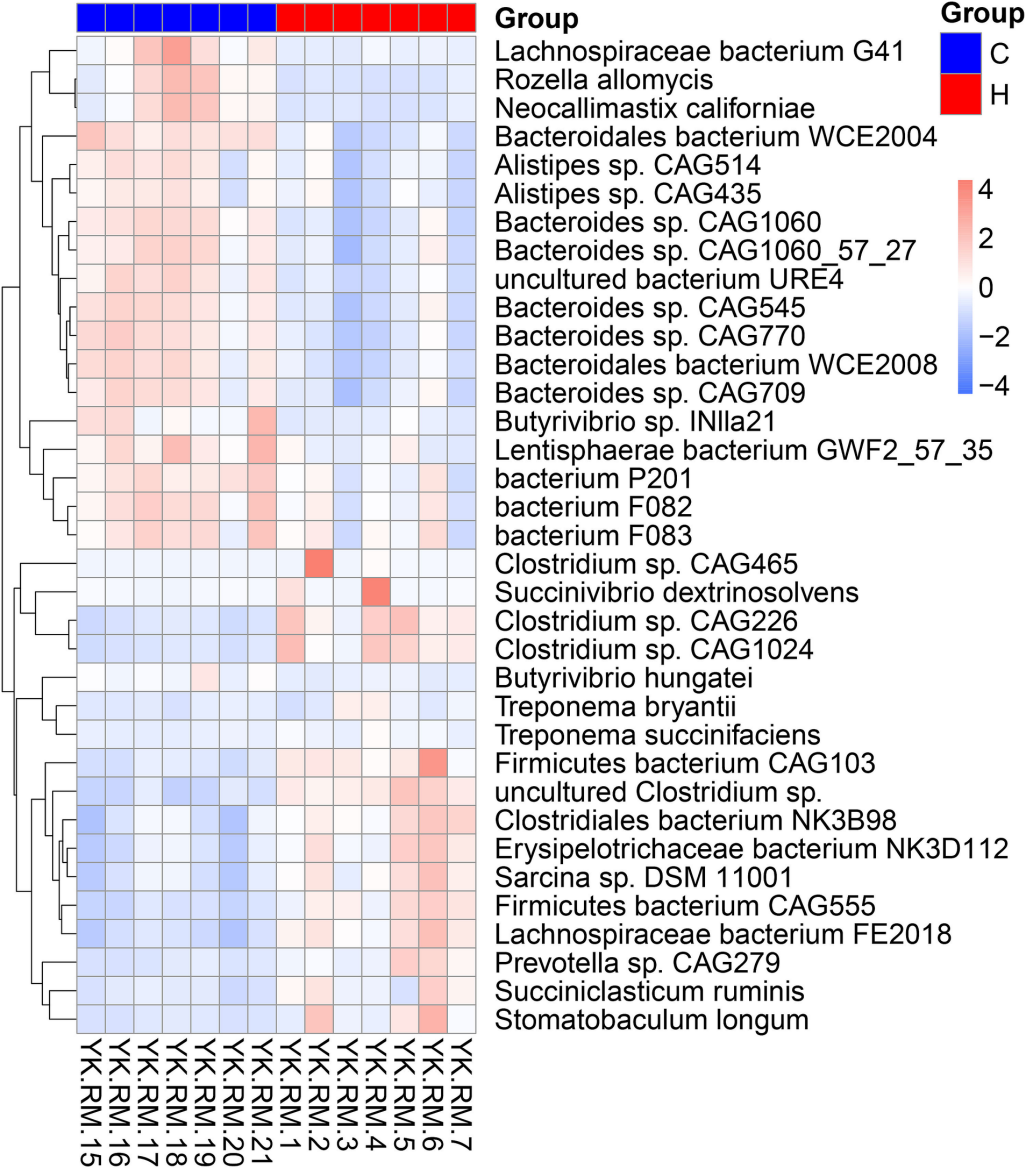
The rumen microbiome differentiating yaks in grazing and housed feeding systems. C group: Yaks traditionally grazed throughout the day on natural pastures. H group: Yaks were raised using a system where they were fed a total mixed ration diet within a house structure.

As expected, the rumen microbiome composition of house-fed yaks significantly differed from that of traditionally fed yaks (C group) (Supplementary Figure S1). Subsequently, we determined the microbial taxonomy associated with the feeding system. The rumen metagenome across all samples comprised 97.56% bacteria, 1.75% eukaryotes, 0.49% archaea, and 0.20% viruses (Supplementary Table S1). The rumen microbial domains, including bacteria and viruses, increased due to the house-feeding system (*p* < 0.05), while the abundance of eukaryotes was higher in the C group, as shown in the KRONA diagram (Figure 1). At the phylum level, the abundance of Firmicutes was greater in the H group compared to the C group, while Bacteroidetes showed decreased abundances in the H group (Figure 1; Supplementary Figure S2). At the genus level, dominant bacteria included *Prevotella*, *Bacteroides*, *Clostridium*, *Selenomonas*, *Alistipes*, *Fibrobacter,* and *Ruminococcus* (Figure 1; Supplementary Figure S3). Higher abundances of genera within bacteria (e.g., *Paraprevotella*, *Bacteroides*, *Alistipes*), fungi (*Piromyces*), and eukaryotes (*Anaeromyces*, *Rhizophagus*, *Rozella*, *Neocallimastix*, *Spizellomyces*, *Batrachochytrium*) were observed in the C group. However, the H group was enriched with additional bacterial genera, including *Streptococcus*, *Bifidobacterium*, *Lachnoclostridium*, *Succinivibrio*, *Bacillus*, *Paenibacillus*, *Mycoplasma*, *Faecalibacterium*, *Ruminococcus*, *Clostridium,* and *Roseburia*. To further investigate differences in rumen microbial species between the two feeding systems, Metastats analysis was conducted (Figure 2). The C group exhibited greater abundances of five *Bacteroides* sp.*, Lachnospiraceae bacterium G41*, *Bacteroidales bacterium WCE2004*, *Rozella allomycis*, *Neocallimastix californiae,* and two *Alistipes* sp. However, *Stomatobaculum longum*, *Succiniclasticum ruminis*, *Prevotella* sp. *CAG279*, 4 *Clostridium* sp., and *Firmicutes bacterium CAG103* were more abundant in the H group.

### The rumen resistome in yaks affected by the housing-feeding system

To investigate the rumen resistome in yaks, metagenomic reads were analyzed against the Comprehensive Antibiotic Resistance Database (CARD), revealing 1,053 Antibiotic Resistance Ontology entries.

ARG abundance in the H group was significantly higher than that in the C group (*p* < 0.05) (Supplementary Figure S4A).

**Figure 3:**
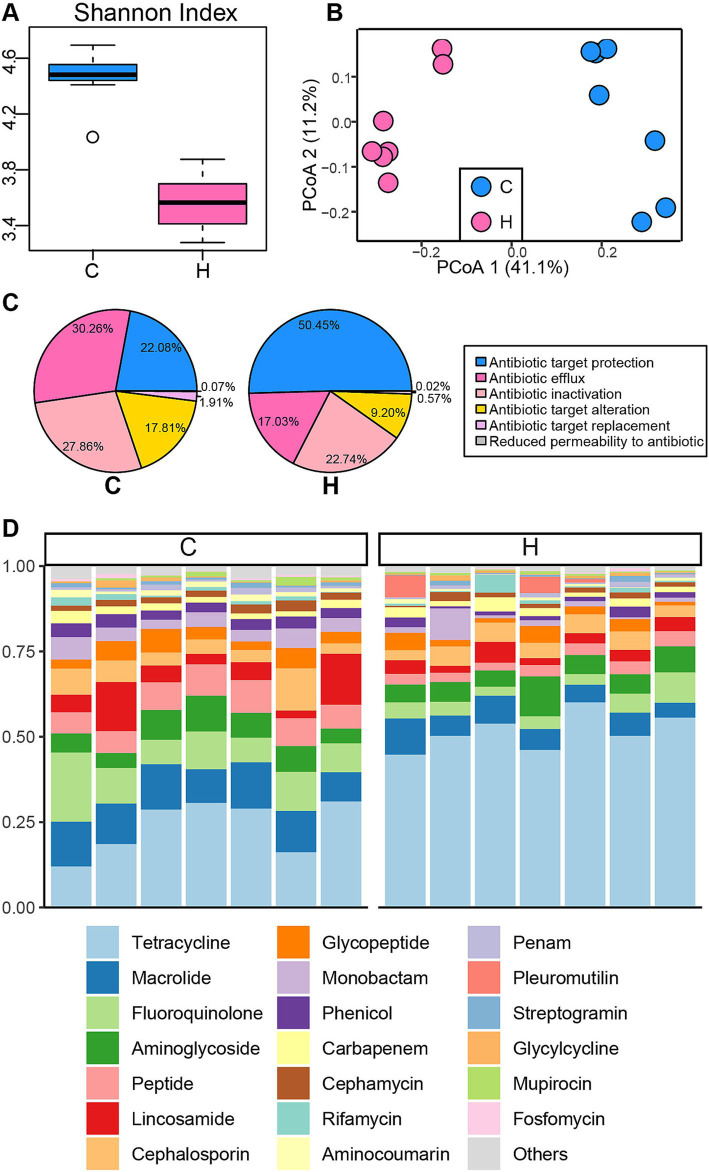
The structure and composition of the rumen resistome in yaks under grazing and house feeding systems. **(A)** Alpha diversity of the rumen resistome. **(B)** Principal Coordinate Analysis (PCoA) of Bray–Curtis distances for the rumen resistome. **(C)** Proportions of antibiotic resistance mechanisms. **(D)** Relative abundance of antibiotic resistance genes (ARGs) at the class level. Each column represents a sample, and each bar represents an ARG class. C group: Yaks traditionally grazed throughout the day on natural pastures. H group: Yaks were raised using a system where they were fed a total mixed ration diet within a house structure.

The Venn plot revealed that the majority of ARGs were shared between the C and H groups, with some unique to each (Supplementary Figure S4B). Subsequently, the Shannon Index of ARGs was calculated (Figure 3A), indicating that the C group had greater ARG diversity compared to the H group (*p* < 0.05). Moreover, a distinct cluster of ARGs was identified between the C and H groups based on Bray-Curtis distance (ANOSIM: R = 0.96, *p* = 0.001) (Figure 3B). Consistently, the composition of ARGs also showed significant differences between the two groups. Antibiotic resistance mechanisms related to antibiotic target protection were the most abundant (C group: 22.08%; H group: 50.45%), followed by antibiotic efflux (C group: 30.26%; H group: 17.03%), antibiotic inactivation (C group: 22.86%; H group: 22.74%), and antibiotic target alteration (C group: 17.81%; H group: 9.2%) (Figure 3C). At the ARG class level, tetracycline was more prevalent in the H group (51.59%) compared to the C group (23.75%), while other ARG classes, including macrolide, fluoroquinolone, peptide, lincosamide, cephalosporin, glycopeptide, monobactam, and phenicol, demonstrated higher relative abundances in the C group (Figure 3D).

**Figure 4:**
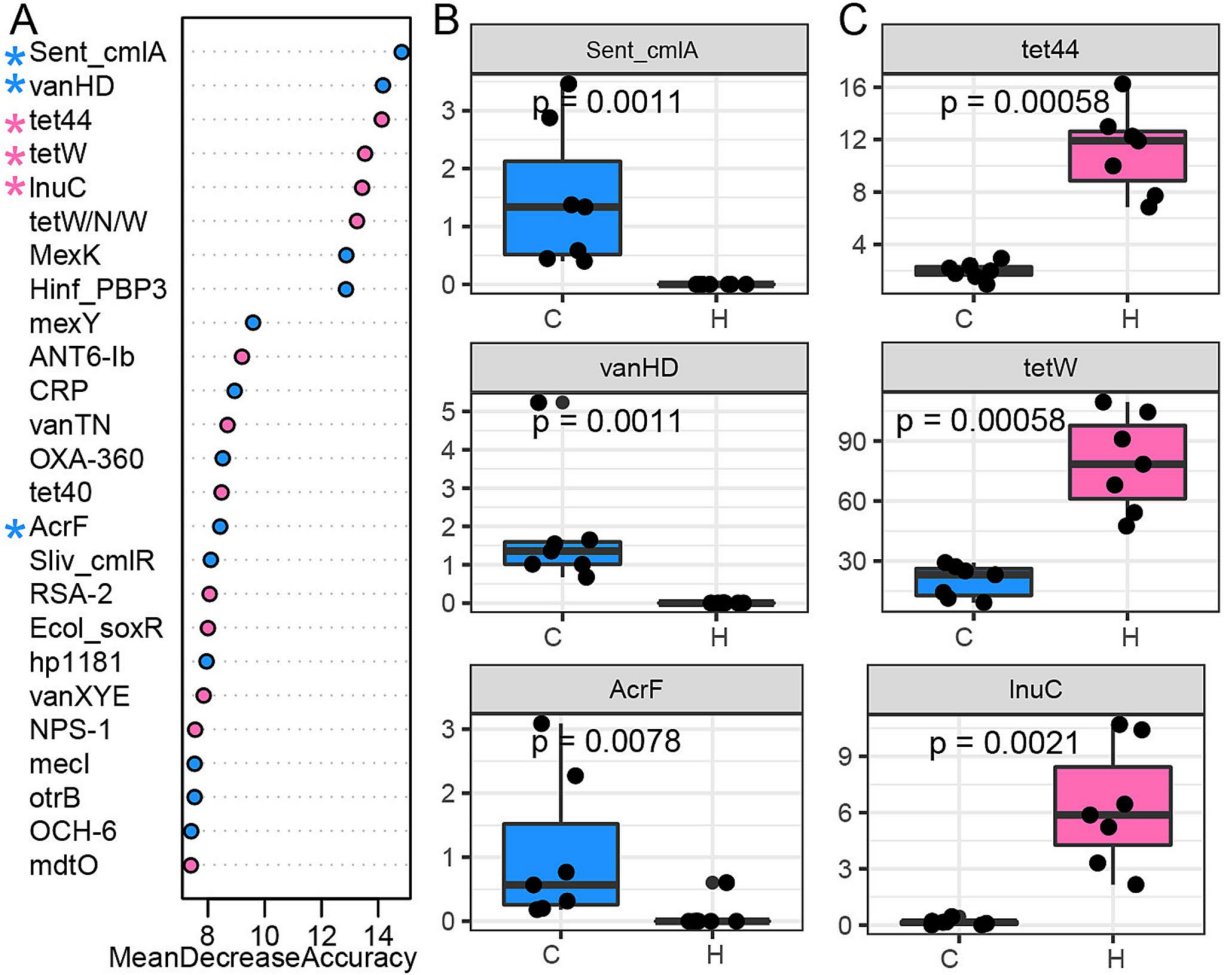
ARG signature differentiates between grazing and house feeding systems. **(A)** A random forest classification model was used to identify the top 25 predictors listed based on their importance score (mean decrease accuracy). **(B)** The signature ARGs were abundant in the rumen of yaks in a natural grazing system. **(C)** The signature ARGs were abundant in the rumen of yaks in a house-feeding system. C group: Yaks traditionally grazed throughout the day on natural pastures. H group: Yaks were raised using a system where they were fed a total mixed ration diet within a house structure.

A random forest classification model was employed to identify ARG signatures that distinguish these two groups. The top 25 most predictive ARGs were ranked based on their mean decrease in accuracy (Figure 4A). ARGs, including Sent_cmlA and Sliv_cmlR (Phenicol), as well as vanHD (Glycopeptide), were enriched in the C group (Figure 4B; Supplementary Figure S5). We also observed that the C group had a higher abundance of MexK, Hinf_PBP3, mexY, CRP OXA-360, AcrF, hp1181, otrB, mecI, and OCH-6, which were shared ARGs across several ARG classes. ARGs associated with tetracyclines, such as tet44, tetW, tetW/N/W, and tet40, were abundant in the H group (Figure 4C; Supplementary Figure S5). Moreover, lnuC (Lincosamide), ANT6-Ib (Aminoglycoside), and two ARGs under Glycopeptide (vanTN, vanXYE) were also abundant in the H group. ARGs (Ecol_soxR, RSA-2, NPS-1, and mdtO) that were shared by several antibiotic classes were also enriched in the H group.

**Figure 5:**
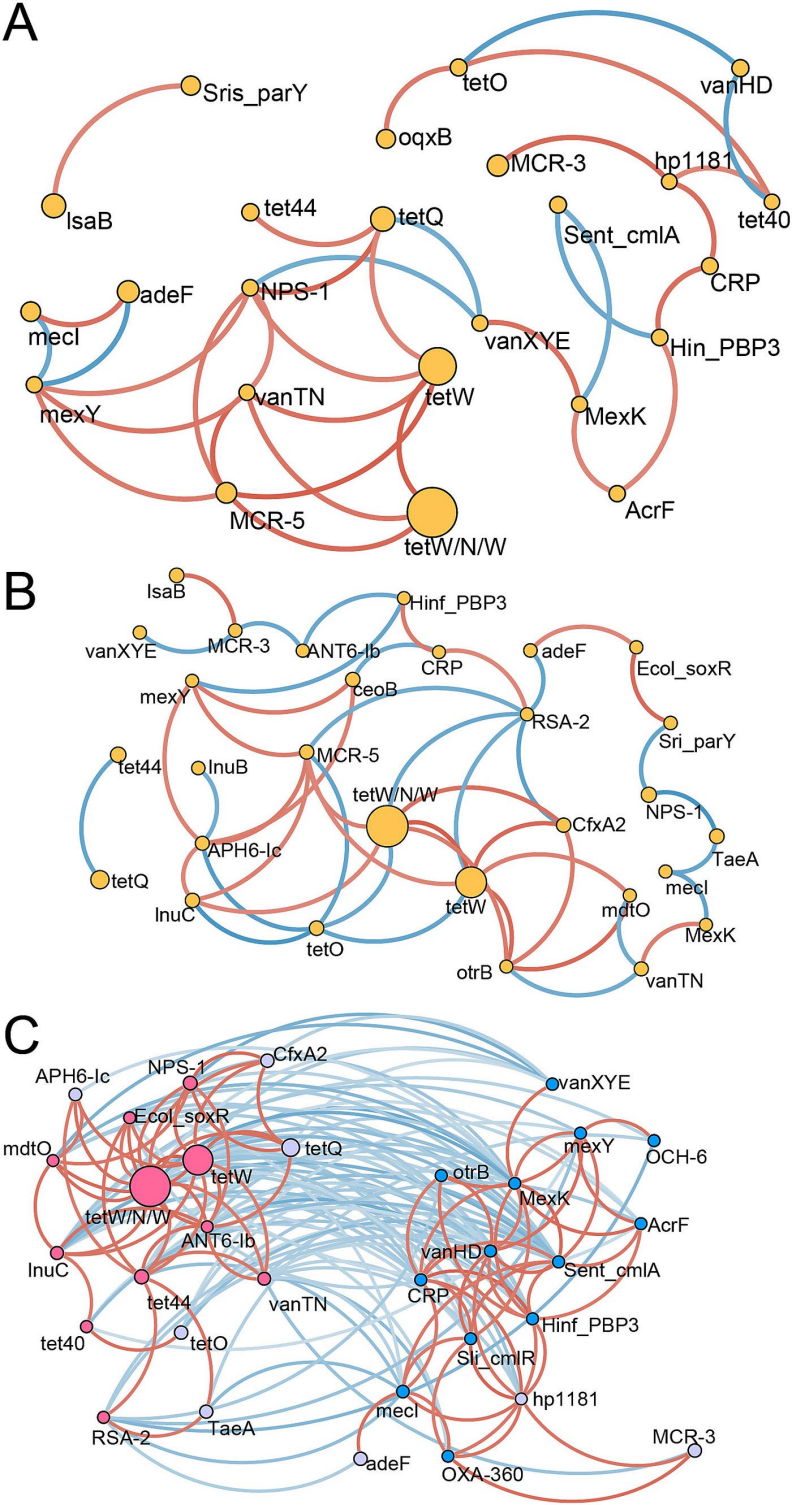
The correlation networks of ARGs. Spearman’s correlation among ARGs in the C group **(A)**, H group **(B)**, and all samples **(C)** is represented. The edge colors (red for positive, blue for negative) indicate the strength of the correlation. Only strong (Spearman’s R > 0.7 or < − 0.7) and significant (*p* < 0.05) correlations are displayed. C group: Yaks traditionally grazed throughout the day on natural pastures. H group: Yaks were raised using a system where they were fed a total mixed ration diet within a house structure.

A network analysis was conducted to reveal the ARG interactions within and between treatment groups (Figure 5). In the C group, ARGs associated with tetracycline demonstrated stronger positive correlations. For example, tetW, tetW/N/W, and tetQ were positively correlated with one another (Figure 5A). Some ARG signatures for the C group identified by the random forest model showed a contrasting pattern, such as the negative correlation between Sent_cmlA and MexK or Hinf_PBP3. However, Hin_PBP3, AcrF, MexK, CRP, and hp1181, which were abundant in the C group, showed positive correlations. In the H group, similar interactions among ARGs of Tetracycline were also observed, including positive correlations between tetW, tetW/N/W, and otrB, along with positive correlations with mdtO and lnuC at higher abundances in the H group (Figure 5B). These results indicated that some consistent ARG interactions were unaffected by the feeding system. Additionally, when all samples were used for network analysis, two distinct subunits with ARG signatures for the C and H groups were observed (Figure 5C), suggesting that alterations in ARGs were caused by the feeding system. ARGs between these two subunits were negatively correlated. Furthermore, positive correlations among ARGs within each subunit were found. For instance, tet44, tetW, tetW/N/W, tet40, lnuC, ANT6-Ib, vanTN, Ecol_soxR, RSA-2, NPS-1, and mdtO were positively related to each other, forming the left subunit, while Sent_cmlA, Sliv_cmlR, vanHD, AcrF, Hinf_PBP3, mexY, CRP OXA-360, MexK, CRP, and hp1181 with positive relationships formed another subunit.

### Rumen microbiome associated with resistome in yaks

**Figure 6:**
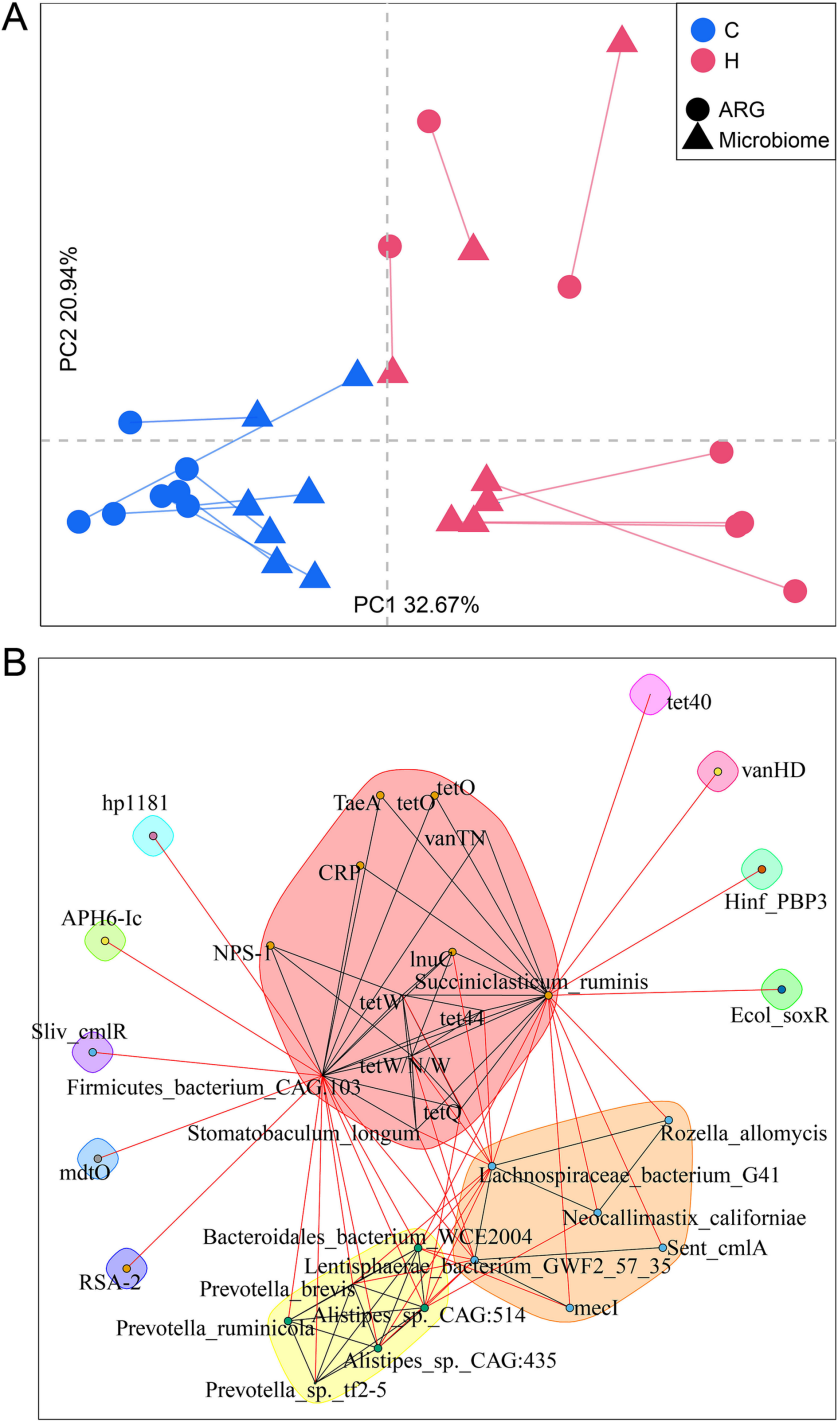
Rumen resistome associated with its bacterial community. **(A)** Procrustes analysis examining the relationship between resistome composition and the microbiome community. **(B)** A network analysis of co-occurrence patterns between ARGs and microbial taxa. The SparCC algorithm was used to calculate the relationships between bacterial taxa and ARGs. C group: Yaks traditionally grazed throughout the day on natural pastures. H group: Yaks were raised using a system where they were fed a total mixed ration diet within a house structure.

Rumen microbiomes, a reservoir of ARGs, are strongly correlated with ARGs (Sabino et al., 2019). In this study, Procrustes analysis revealed a significant correlation between the composition of rumen microbial communities and ARG profiles (correlation coefficient *r* = 0.9249; *p* = 0.0001) (Figure 6A). Moreover, the feeding regime drove this correlation, as significant clusters were observed between these two groups.

To thoroughly explore the correlation between the microbiome and resistome, we next aimed to analyze the co-occurrence patterns between ARGs and major microbial species using network analysis (Figure 6B). High abundances of *Stomatobaculum longum*, *Succiniclasticum ruminis,* and *Firmicutes bacterium CAG103* in the H group were strongly correlated with the ARG signatures for the H group, such as tet44, tetW, tetW/N/W, tet40, lnuC, and vanTN. Interestingly, two clusters with high abundances of features (either ARGs or microbiota) in the C groups were observed. Sent_cmlA and mecI were related to *Lachnospiraceae bacterium G41*, *Rozella allomycis*, *Neocallimastix californiae,* and *Lentisphaerae bacterium GWF2_57_35*, forming one subunit. Another cluster included strongly associated bacteria, including *Bacteroidales bacterium WCE2004*, *Alistipes* sp. *CAG514*, *Alistipes* sp. *CAG435,* and three *Prevotella* sp. In addition, *Succiniclasticum ruminis* and *Firmicutes bacterium CAG103* were critical nodes connecting these clusters, suggesting their importance in carrying ARGs.

### Phylogeny of ARGs related to rumen microbial feeding regimes

Assigning microbial taxonomy to metagenomic reads containing ARGs can help identify the microbiome of ARG carriers in the yak rumen. The bacterial phyla associated with ARG carriers included Bacteroidetes, Firmicutes, and Proteobacteria (Supplementary Figure S6). A greater number of Proteobacteria carrying ARGs were observed in the C group, while Firmicutes were the ARG hosts in the H group. At the family level, the main ARG carriers in the C group were *Ruminococcaceae, Yersiniaceae, Pseudomonadaceae, Rikenellaceae, Enterobacteriaceae, Cellulomonadaceae, and Rhizobiaceae*. In contrast, the H group showed a higher abundance of *Lachnospiraceae*, *Clostridiaceae*, *Selenomonadaceae*, *Fibrobacteraceae*, *Succinivibrionaceae,* and *Flavobacteriaceae* (Supplementary Figure S7). Moreover, the major species carrying ARGs included *Firmicutes bacterium CAG:103*, *Prevotella ruminicola*, *Bacteroidales bacterium WCE2004, Clostridiales bacterium,* and *Prevotella* sp. *BP1-145,* which exhibited different relative abundances between these two groups (Supplementary Figure S8).

**Figure 7:**
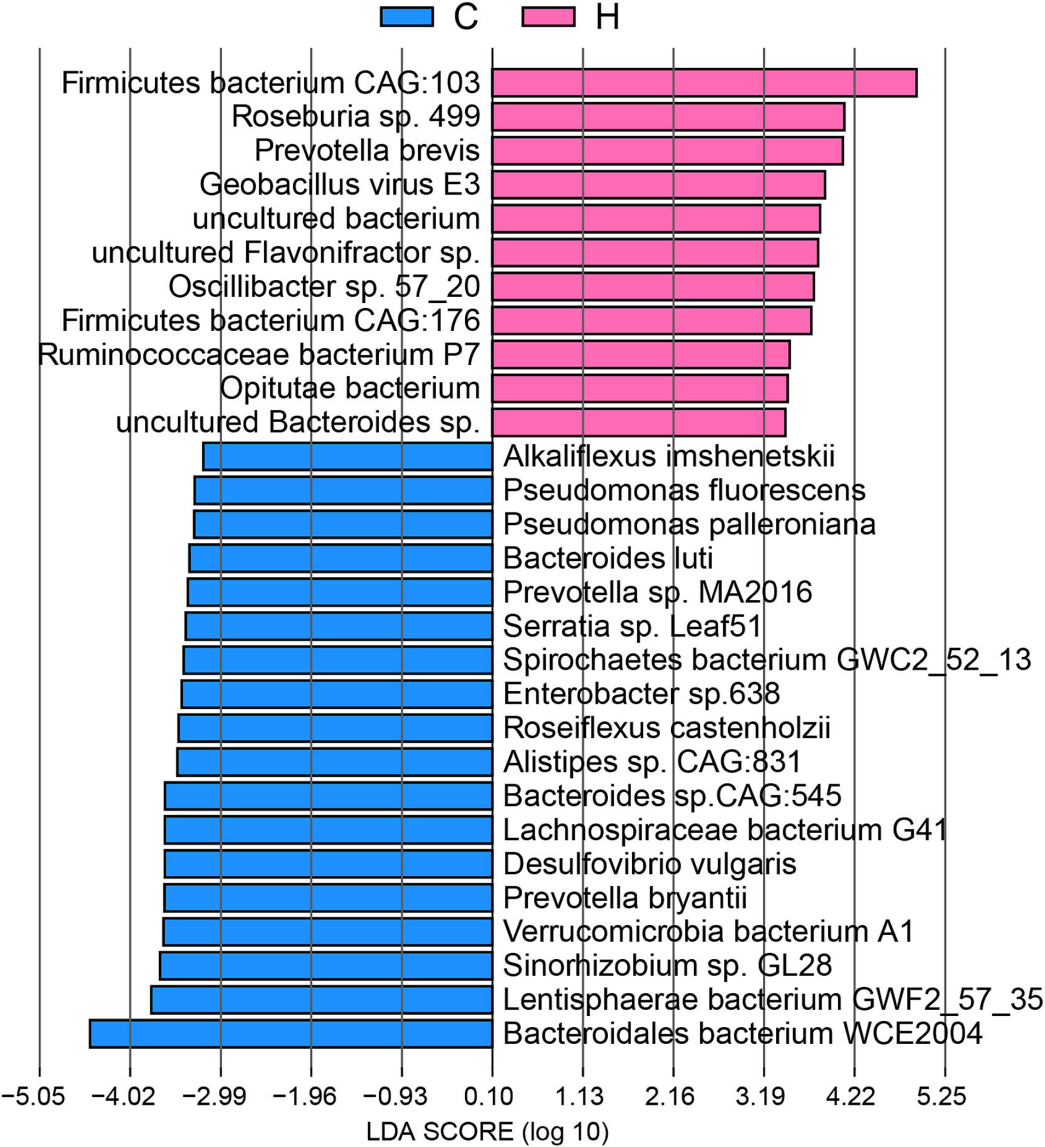
The signature microbiome of ARG carriers, distinguishing between grazing and house feeding systems. C group: Yaks traditionally grazed throughout the day on natural pastures. H group: Yaks were raised using a system where they were fed a total mixed ration diet within a house structure.

Thus, to thoroughly analyze the origins of ARGs from the bacterial species affected by feeding systems, we performed LEfSe (Figure 7). Our findings indicated that several abundant bacteria in the rumen community of C group yaks were key ARG carriers, including *Bacteroidales bacterium WCE2004*, *Lentisphaerae bacterium GWF2_57_35,* and *Lachnospiraceae bacterium G41*. Notably, other microbiota, such as *Sinorhizobium* sp. *GL28*, *Verrucomicrobia bacterium A1*, *Prevotella bryantii*, *Desulfovibrio vulgaris*, *Bacteroides* sp. *CAG:545*, *Alistipes* sp. *CAG:831*, *Roseiflexus castenholzii*, *Enterobacter* sp.*638*, *Spirochaetes bacterium GWC2_52_13*, *Serratia* sp. *Leaf51*, *Prevotella* sp. *MA2016*, *Bacteroides luti*, *Pseudomonas palleroniana*, *Pseudomonas fluorescens*, and *Alkaliflexus imshenetskii*, served as major hosts for ARGs in the C group. In contrast, the abundant *Firmicutes bacterium CAG:103* was the primary carrier of ARGs in the H group. Other ARG carriers included *Roseburia* sp. *499*, *Prevotella brevis*, *Geobacillus virus E3*, *uncultured Flavonifractor* sp., *Oscillibacter* sp. *57_20*, *Firmicutes bacterium CAG:176*, *Ruminococcaceae bacterium P7,* and *Opitutae bacterium*.

### Rumen ARGs and their bacterial carriers associated with feeding regime

**Figure 8:**
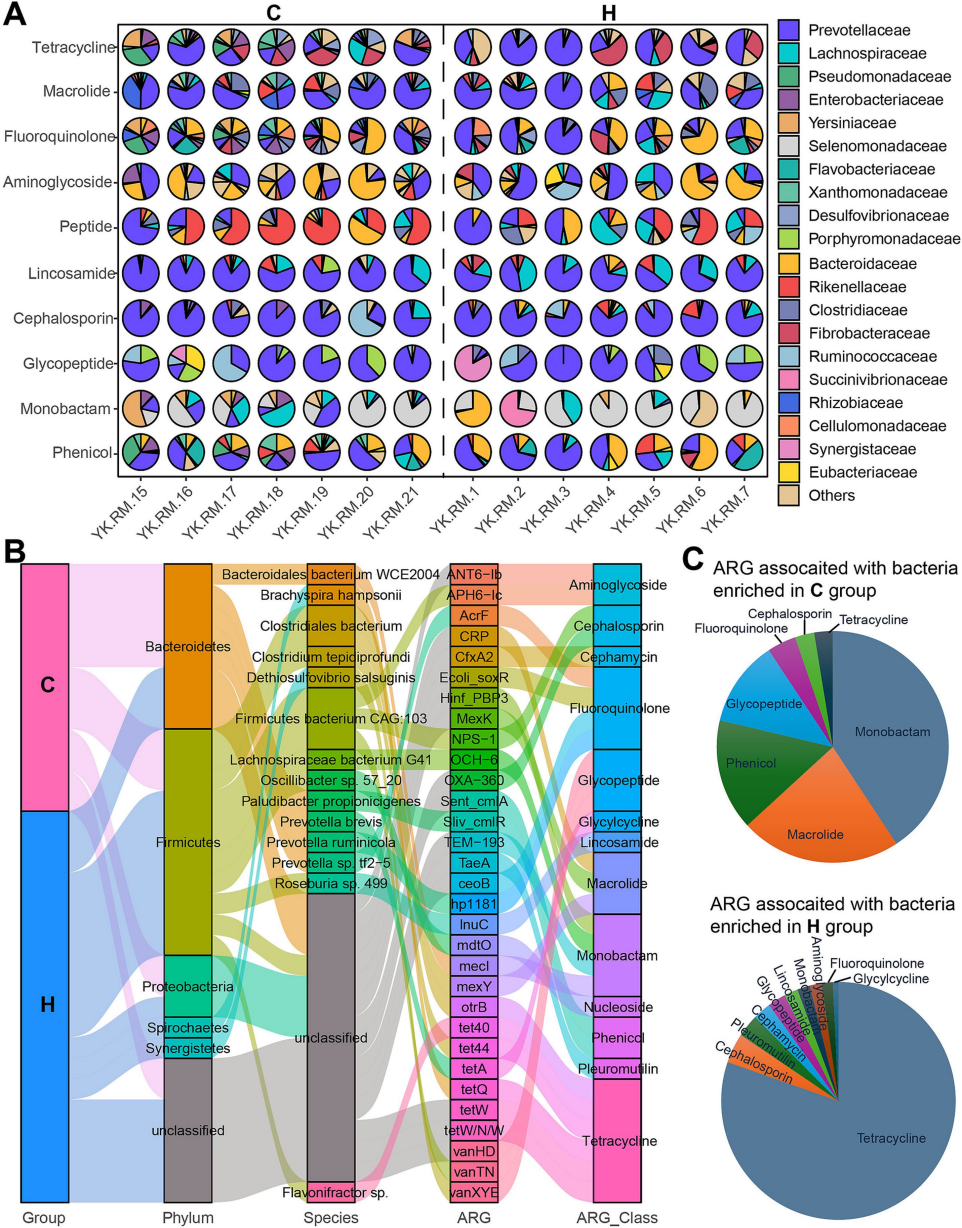
Microbiome species contributing to the rumen resistome. **(A)** The contribution of the top 20 microorganisms at the family level to antibiotics across various samples. **(B)** A Sankey plot illustrates the connections between the differential microbes from C and H (1st column) with ARGs (3rd column) and antibiotic class (4th column). **(C)** Fan plots depict the type and proportion of antibiotics associated with bacteria that are significantly enriched in the C group (top) and the H group (left). C group: Yaks traditionally grazed throughout the day on natural pastures. H group: Yaks were raised using a system where they were fed a total mixed ration diet within a house structure.

To determine whether specific antibiotics were contributed by certain bacteria, a functional contribution mapping analysis was conducted (Figure 8A). Variations in microbial contributions to antibiotics, such as fluoroquinolone, were observed among the top ARG classes. Additionally, the effects of the feeding system were observed. For example, the primary contributors to tetracycline in the C group were *Yersiniaceae*, *Prevotellaceae,* and *Desulfovibrionaceae*, while *Prevotellaceae*, *Brachyspiraceae,* and *Fibrobacteraceae* were the major contributors in the H group. Moreover, shared microbial contributors were identified. For Macrolide, Lincosamide, Cephalosporin, and Glycopeptide, *Prevotellaceae* served as the primary carrier in both groups. In addition, for aminoglycosides, although individual variations were observed, resulting in no differences between these two groups, *Bacteroidaceae* and *Prevotellaceae* remained the two main bacterial hosts.

Subsequently, we further analyzed the link between microbial species and ARGs. In the C group, the *Bacteroidales bacteria WCE2004*, including *Paludibacter propionicigenes*, *Prevotella brevis*, *Prevotella ruminicola,* and *Prevotella* sp. *tf2-5* (Bacteroidetes phylum), harbored mecI, Sliv_cmlR, AcrF, and hp1181 (Figure 8B). Other significant ARG carriers, such as *Clostridiales bacterium* and *Lachnospiraceae bacterium G41* (Firmicutes), exhibited a high abundance of mexY, otrB, and OCH-6 within the C group. In the H group, members of Firmicutes, including *Clostridium tepidiprofundi*, *Firmicutes bacterium CAG:103*, *Oscillibacter* sp. *57_20*, *Roseburia* sp. *499,* and *uncultured Flavonifractor* sp., displayed their ARG signatures, which included tet44, vanXYE, NPS-1, vanTN, mdtO, lnuC, tetW/N/W, tetW, Ecoli_soxR, and tet40. Among the ARGs associated with these bacteria, most were linked to Monobactam, Macrolide, Phenicol, and Glycopeptide in the C group, while tetracycline was abundant in the H group (Figure 8C).

## Discussion

The spread of ARGs poses a threat to public health and the farming environment, becoming a hotspot for ARG reservoirs (Mahmud et al., 2024). Previous studies have mainly focused on how ARGs in animals spread to surrounding natural ecosystems, farm workers, and consumers of animal products (Ding et al., 2022; Kim and Ahn, 2022; Mahmud et al., 2024). However, the influence of a new feeding system on the animal resistome remains unclear. In this study, we analyzed rumen ARGs in yaks under a conventional rearing (traditional grazing) system to compare the resistome of yaks receiving a house feeding system. This comparison revealed the environmental effects of feeding on the animal ARG composition in a high-altitude area. Under the conventional rearing system, yaks graze annually on natural pastures without any artificial interventions, such as dietary supplementation or access to farmworkers or veterinarians, while yaks reared in a house feeding system receive a nutritionally designed diet, live in a small pen, and have more opportunities to interact with farm workers.

Based on metagenomics, we found that the yak rumen microbiome and the composition of ARGs were influenced by the housing feeding system. This feeding method enriched certain ARGs, such as tetracyclines.

ARG interactions depended on the feeding system, with signature ARGs in each group exhibiting specific correlations, as identified by the random forest model. Moreover, our results confirmed a strong association between the microbiome and the resistome. Additionally, the microbiome carrying signature ARGs did not solely differentiate these two feeding systems, indicating that factors beyond diet may also contribute to changes in the rumen microbiota and resistome in yaks.

Compared to grazing yak, house-fed yaks exhibited a distinct rumen microbial composition due to their high-grain diet (Dai D. et al., 2023; Huang et al., 2021; Xu et al., 2021). However, previous studies relied on 16S rRNA sequencing technology, which is limited in its ability to identify alterations at the microbial species level. In our study, we employed rumen metagenome to uncover a broader range of microbial species associated with the house feeding system.

Except for bacterial changes, we also observed alterations in rumen eukaryotes and viruses, likely influenced by the nutritional composition of the house-fed diet. Grazing yaks exhibited greater abundances of *Bacteroides* sp. (five species), *Rozella allomycis*, and *Alistipes* sp. (two species), which may be due to their preferred roles in fiber-based polysaccharides digestion (Guo et al., 2021; Sista Kameshwar and Qin, 2019; Xue et al., 2022). However, the enrichment of *Stomatobaculum longum* and *Succiniclasticum ruminis* in house-fed yaks may be associated with VFA production from a high-grain diet (Petri et al., 2020). Additionally, the increased abundance of *Clostridium* sp., which is involved in amino acid and carbohydrate metabolism, suggests an enhanced ability to utilize protein and starch in the house feeding system (Hu D. et al., 2021). Overall, dietary nutrients appear to be the primary factor driving microbial changes in house-fed yaks.

Yaks are a good model for classifying how a housed feeding system alters the gut resistome, possibly because traditionally reared yaks graze on natural pasture, providing ample living space, a high fiber intake, and reduced contact with farmworkers and antibiotics. However, a housed feeding system significantly modifies these feeding strategies. Reports have indicated that rumen resistome profiles are influenced by diet (Auffret et al., 2017). This study found significant alterations in ARG structures and compositions between these two feeding systems, suggesting that a house-feeding system, especially the dietary factor, could induce changes in the rumen resistome. Tetracycline was predominant in the rumen of house-fed yaks. These results indicate that house feeding may simplify the dominant ARGs due to increased artificial interventions, whereas grasses in natural pastures are influenced by various environmental factors. A previous study also found that a high-grain diet enriched rumen tetracycline (Mu et al., 2021).

Tetracycline is a class of broad-spectrum antibiotics characterized by a phenanthrene parent nucleus and produced by certain species of *Streptomyces* (Zhuang et al., 2024). It has been widely used in both human and veterinary medicine to treat bacterial infections and to promote the growth and feed efficiency of livestock (Daghrir and Drogui, 2013). In the current study, tet44, tetW, tetW/N/W, and tet40 within tetracycline were found to be enriched in house-fed yaks, which could be due to feeding strategies or human interactions. Other ARGs, such as lnuC (Lincosamide), were also enriched in the rumen of yaks under house feeding systems. A previous study confirmed that lincosamide members (mef(En2) and lnu(AN2)) could transfer between cows and farmworkers (Mahmud et al., 2024). It is speculated that yaks may acquire ARGs from farm workers or veterinarians due to the interactions involved in feeding and handling.

A previous study reported that ARGs with higher herd prevalence are associated with increasing animal density (Markland et al., 2019), which aligns with the findings of this study. Correspondingly, the lower ARG count found in the rumen of grazing yaks is likely due to the lower herd density in natural pastures. Moreover, we found that macrolide, fluoroquinolone, peptide, lincosamide, cephalosporin, glycopeptide, monobactam, and phenicol resistance genes were more abundant in the rumen of grazing yaks ARGs. Specifically, Sent_cmlA and Sliv_cmlR (phenicol) and vanHD (glycopeptide) were enriched in the C group (Figure 4B; Supplementary Figure S5). A previous study confirmed the spread of ARGs between pasture soil and grazing cattle (Mhuireach et al., 2022), suggesting that the rumen resistome of grazing yaks may acquire ARGs from pasture environments. Therefore, the feeding systems, including factors of diet, feeding environment, and even farmworker contacts, could modulate the rumen resistome.

A deep understanding of ARG interactions may explain how various factors shape the rumen resistome. Our results show that ARGs associated with tetracycline were positively correlated regardless of the feeding system, suggesting strong coordination among some ARGs. Moreover, although some ARGs may exhibit negative correlations, they remain unaffected by the feeding system, suggesting that feeding systems primarily alter core ARGs that shape the resistome composition within a community.

While researchers have attempted to elucidate the molecular mechanisms underlying ARG interactions, the complex relationships between ARGs and their host carriers remain poorly understood (Darby et al., 2023). In the future, a thorough investigation of resistome assembly and interactions within a community is urgently needed.

Our study as well as a previous study found a strong correlation between the rumen microbiome and the resistome (Chai et al., 2024b), suggesting that alterations in rumen bacterial profiles lead to changes in the resistome. This is reasonable, as the microbiome serves as the primary carrier of ARGs (Li et al., 2021). Specifically, the rumen of yaks in a housed feeding system exhibited a higher abundance of *Stomatobaculum longum*, *Succiniclasticum ruminis,* and *Firmicutes bacterium CAG103,* which were correlated with its signature ARGs (e.g., tet44, tetW, tetW/N/W, tet40, lnuC, and vanTN).

A similar correlation pattern between the microbiome and resistome was also observed in the rumen of grazing yaks. The genomes of ruminal bacteria carrying ARGs are typically classified, with tetW being the most abundant gene in the rumen. This gene was detected in 28 bacterial genomes with highly similar nucleotide sequences (Darby et al., 2023). A unique modular pattern of integrative and conjugative elements (ICE) in the bacterial genome is associated with the tetW gene. The ICE structure is essential for conjugation, recombination, and regulation, facilitating the horizontal transfer of tetW between bacteria. Further studies are needed to investigate the deeper associations between the rumen microbiome and ARGs.

Bacteroidetes, Firmicutes, and Proteobacteria phyla were dominant carriers of ARGs in the rumen (Chai et al., 2024b; Sabino et al., 2019). In the rumen of grazing yaks, bacterial species carrying ARGs included *Bacteroidales bacterium WCE2004* (Bacteroidetes phylum) and *Lachnospiraceae bacterium G41* (Firmicutes phylum), along with numerous members of Proteobacteria such as *Sinorhizobium* sp. *GL28*, *Pseudomonas palleroniana*, *Pseudomonas fluorescens,* and *Alkaliflexus imshenetskii*. Bacterial members of Proteobacteria were found to increase from non-grazing to grazing diets, making them a major host of ARGs (Belanche et al., 2019; Chai et al., 2024b). Therefore, it is not surprising that grazing yaks may access various sources of ARGs in a natural pasture. The relatively harsh environment for grazing yaks may contribute to the increased abundance of Proteobacteria (e.g., *Pseudomonas*). In contrast, the rumen of yaks on a housed feeding system was primarily composed of *Firmicutes bacterium CAG:103*, *Roseburia* sp. *499,* and *Clostridiales bacterium* from Firmicutes, as well as species of Bacteroidetes, such as *Bacteroidales bacterium WCE2004, Prevotella ruminicola*, and *Prevotella* sp. *BP1-145*.

The dietary effects of the housed feeding system on ARG carriers were also observed, with more species from the Firmicutes and Bacteroidetes phyla—both associated with nutrient digestion—classified as ARG carriers. Thus, feeding systems and living environments affect rumen microbial composition and, consequently, the resistome.

One limitation of this study is that the resistome in the diet, environment, and humans was not investigated, resulting in a gap in understanding ARG transmission among communities. Future studies should examine the resistome of source environments that may influence the rumen ARG community. Another limitation of this study is the small sample size; however, the significant results between treatments indicate a strong confidence level in our data. In the future, a study with a larger sample size or alternative methods should be implemented to better understand the mechanisms of ARG spread in yaks.

## Conclusion

Our study confirmed that the house feeding system significantly altered the rumen microbiome and resistome compared to traditional grazing practices in yaks. We identified key signature features of the rumen microbiome and ARGs, providing insights into how the feeding system and environment affect the composition of yak rumen ARGs. The house feeding system led to an increased abundance of tetracycline resistance genes in the rumen, a concerning trend given that intensive farming uses tetracycline as both an infection preventer and a growth promotor. While the effects of the feeding system and individual variation influenced ARG interactions, some stable correlations among ARGs remained unaffected. The rumen resistome was strongly associated with its microbiome, and the primary species contributing to ARGs were identified, suggesting that modulation of specific microbiotas could help regulate the rumen resistome in yaks. Overall, factors associated with the house feeding system—including diet, living environments, and farmworker contacts—contributed to changes in the rumen resistome of yaks living in the high-altitude regions.

## Data Availability

Sequencing data generated from shotgun metagenomes in this study have been deposited with the NCBI SRA (PRJNA795434) and are publicly available.

## Footnotes

^1^ http://galaxy.biobakery.org/
