# Supplementary material for: Feeding systems influence the rumen resistome in yaks by changing the microbiome
Source: Front Microbiol. 2025 Mar 19;16:1505938. doi: 10.3389/fmicb.2025.1505938 (PMC11961883; doi:10.3389/fmicb.2025.1505938)

Table S1 Rumen microbial domains in grazing and intensive yaks

|  | C | H | *P* value |
| --- | --- | --- | --- |
| Bacteria | 97.05% | 98.06% | 0.043 |
| Eukaryota | 2.41% | 1.09% | 0.017 |
| Archaea | 0.37% | 0.62% | 0.053 |
| Viruses | 0.17% | 0.23% | 0.017 |

Figure S1 Beta diversities of rumen microbiome


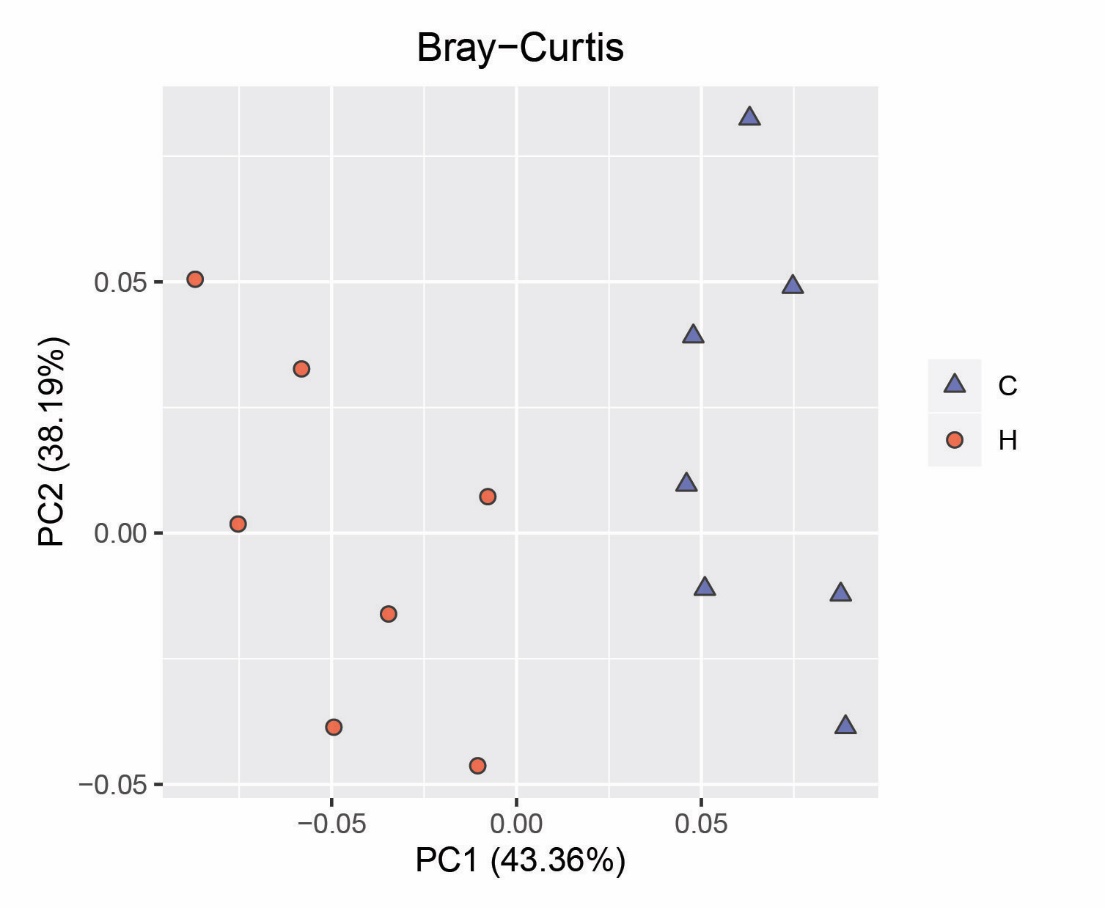


Figure S2 Profiles of rumen microbial composition at the phylum level


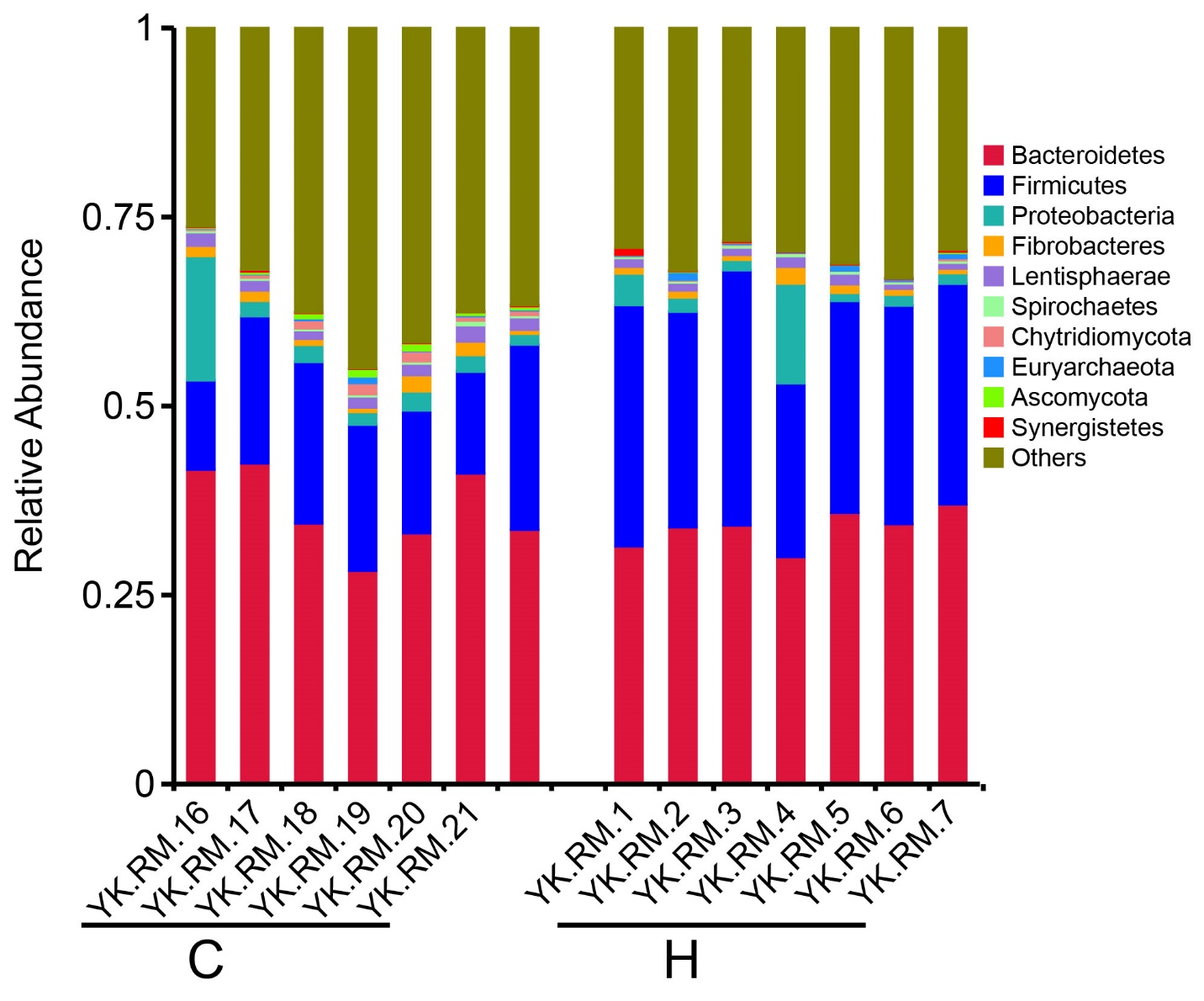


Figure S3 Profiles of rumen microbial composition at the genus level


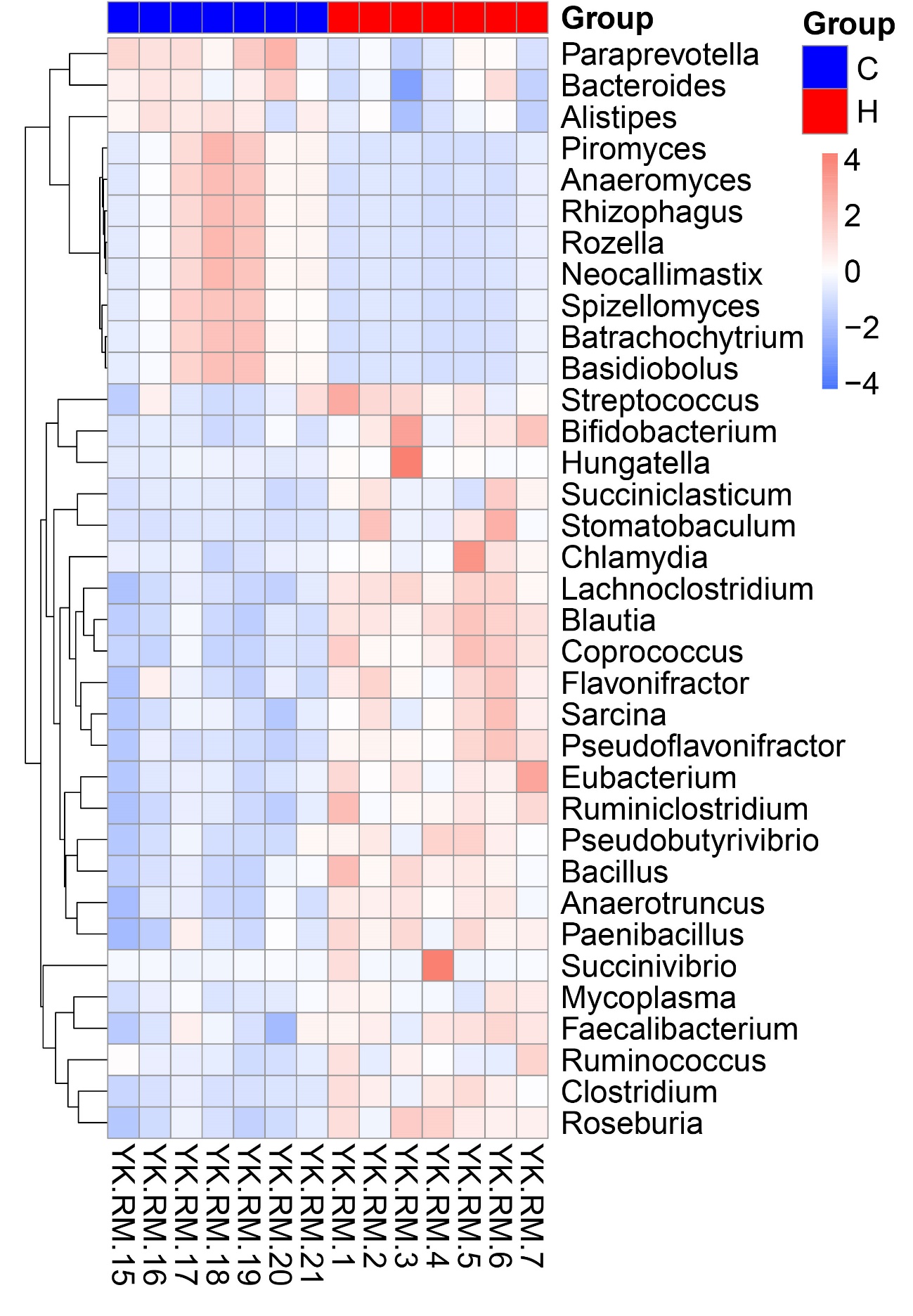


Figure S4 Different ARGs in two feeding system


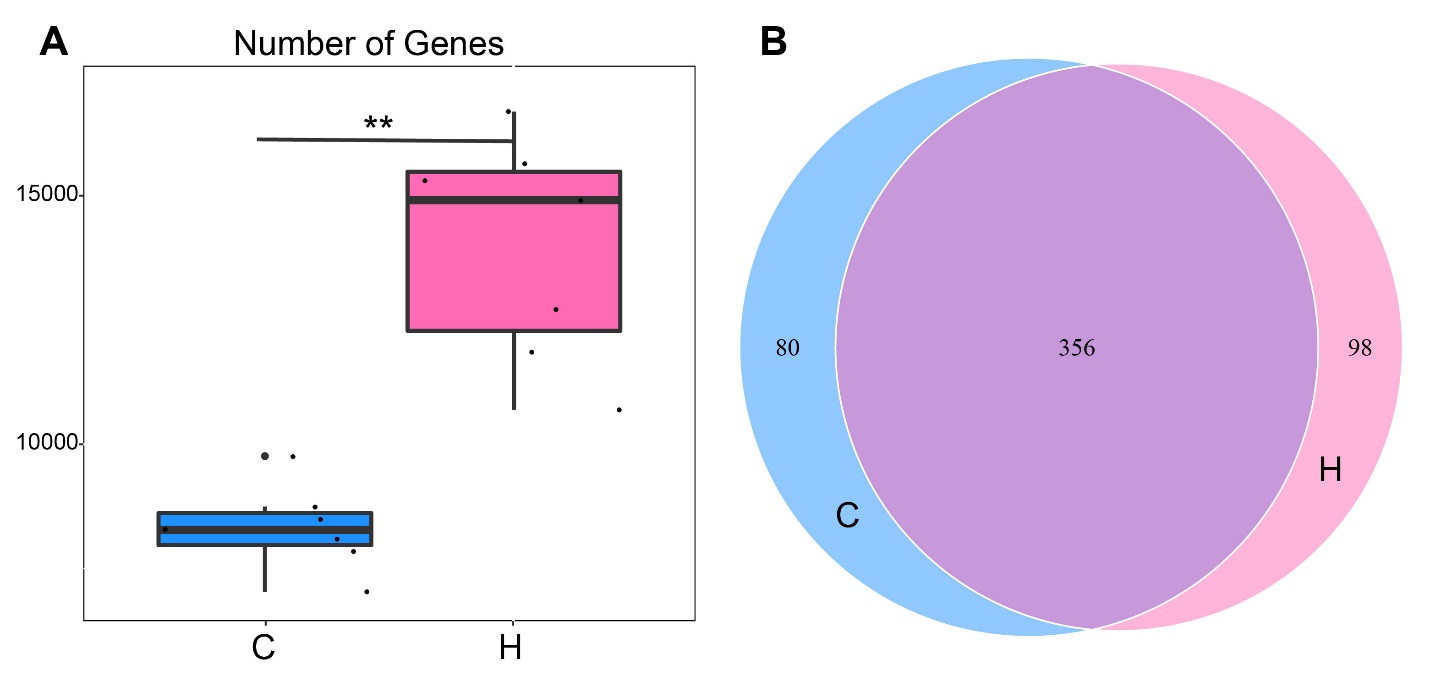


Figure S5 Boxplots of rumen signature ARGs identified by random forest algorithm


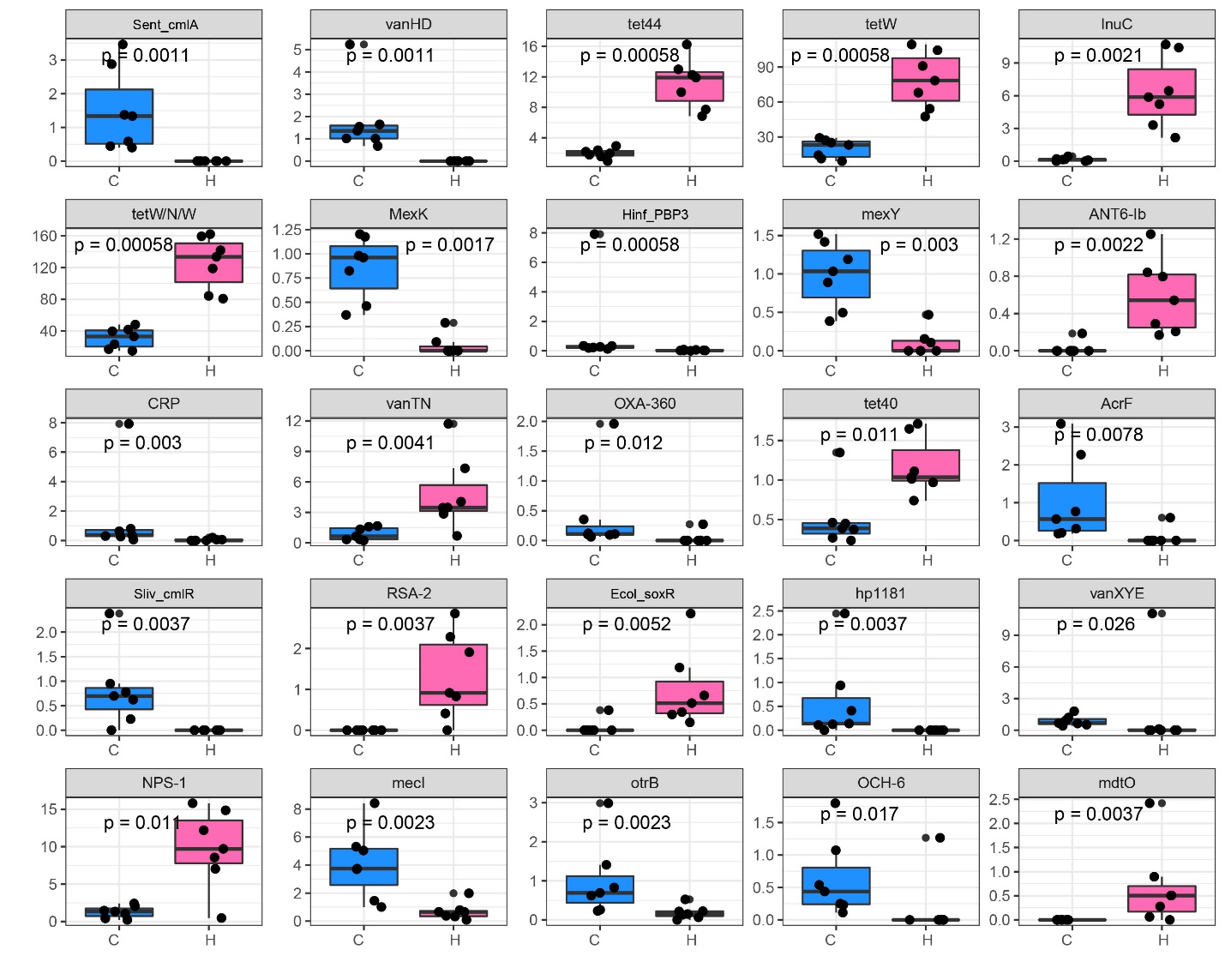


Figure S6 Composition of rumen microbiota of ARG carriers at the phylum level


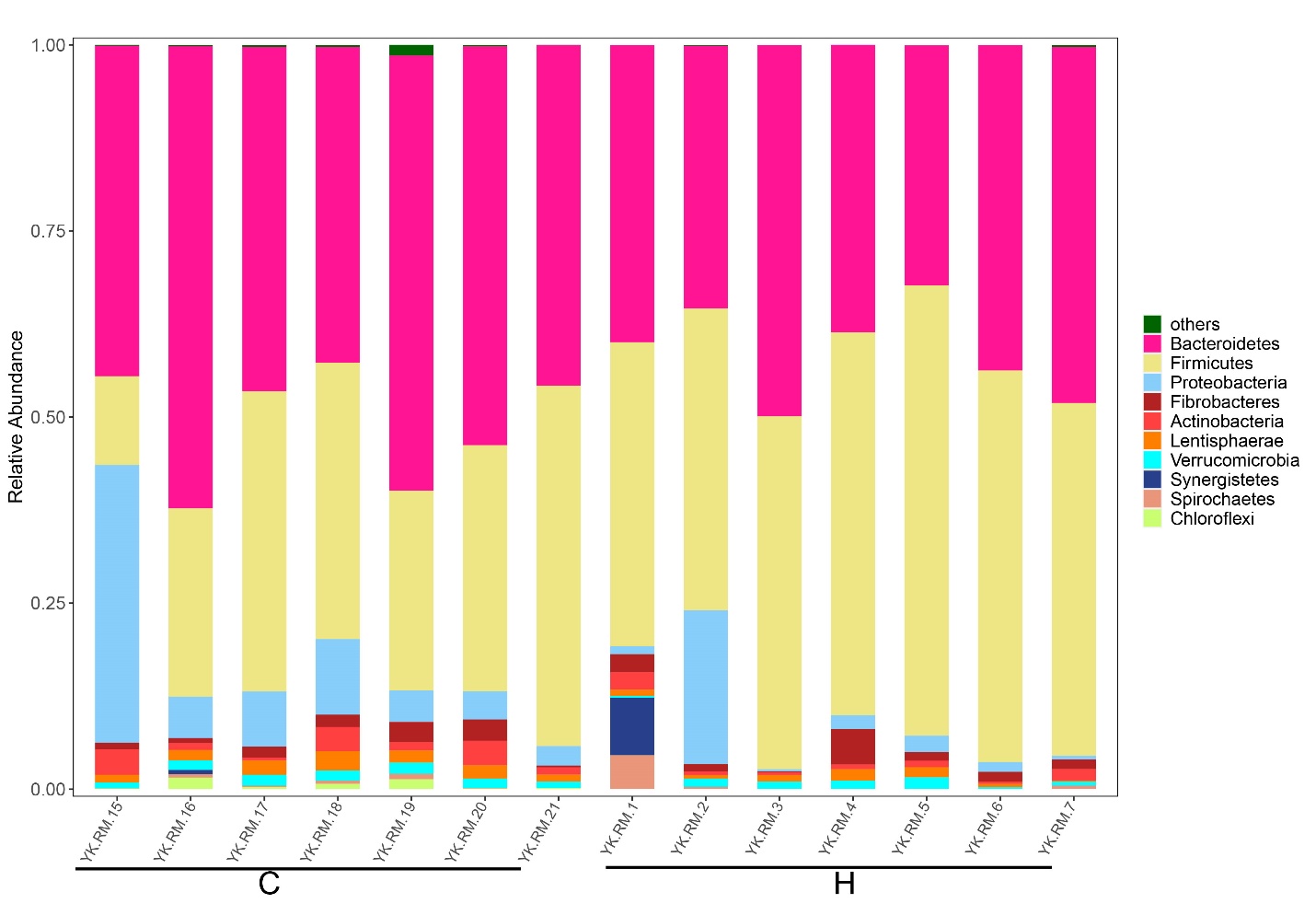


Figure S7 Composition of rumen microbiota of ARG carriers at the family level


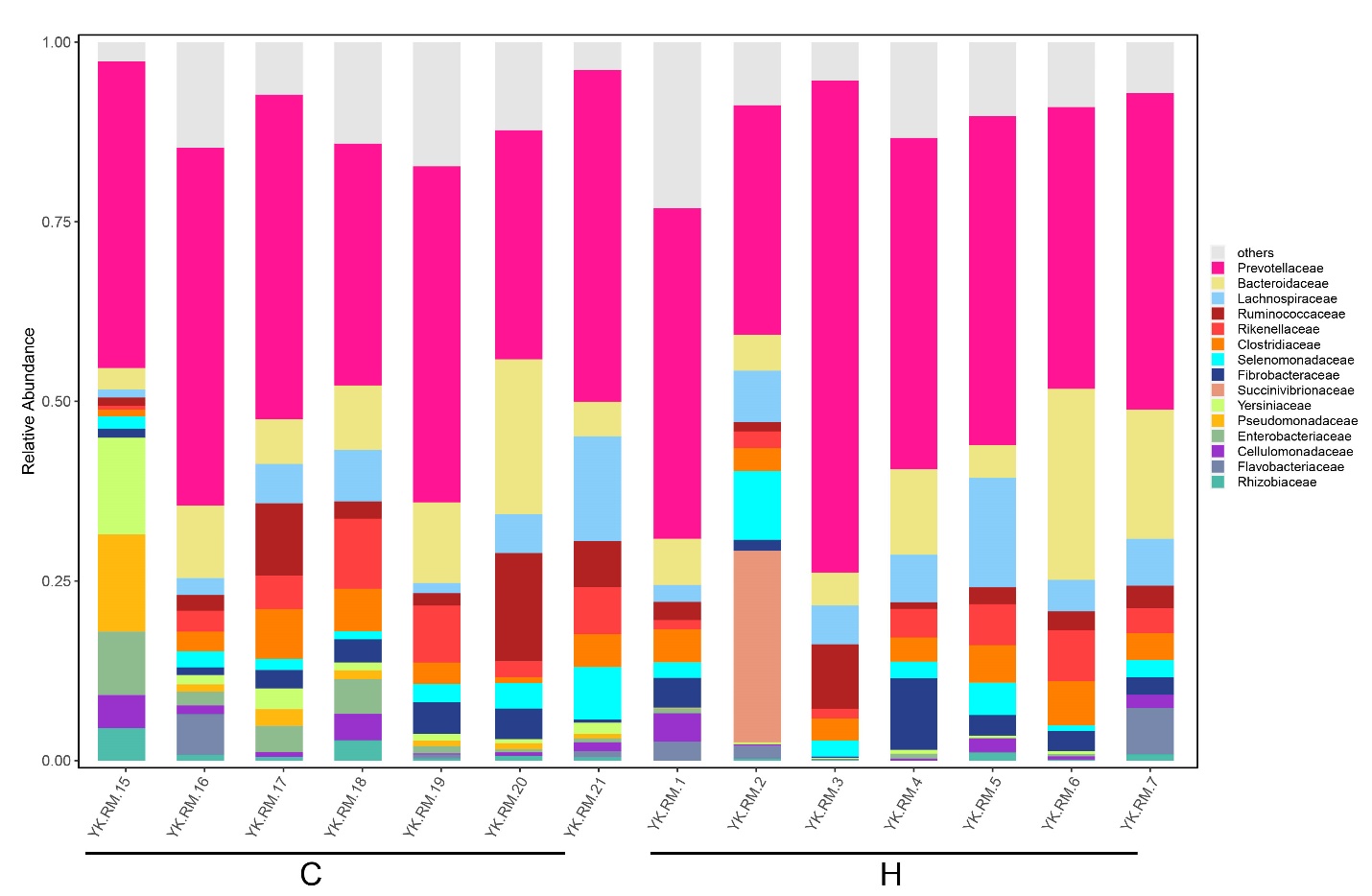


Figure S8 Composition of rumen microbiota of ARG carriers at the species level


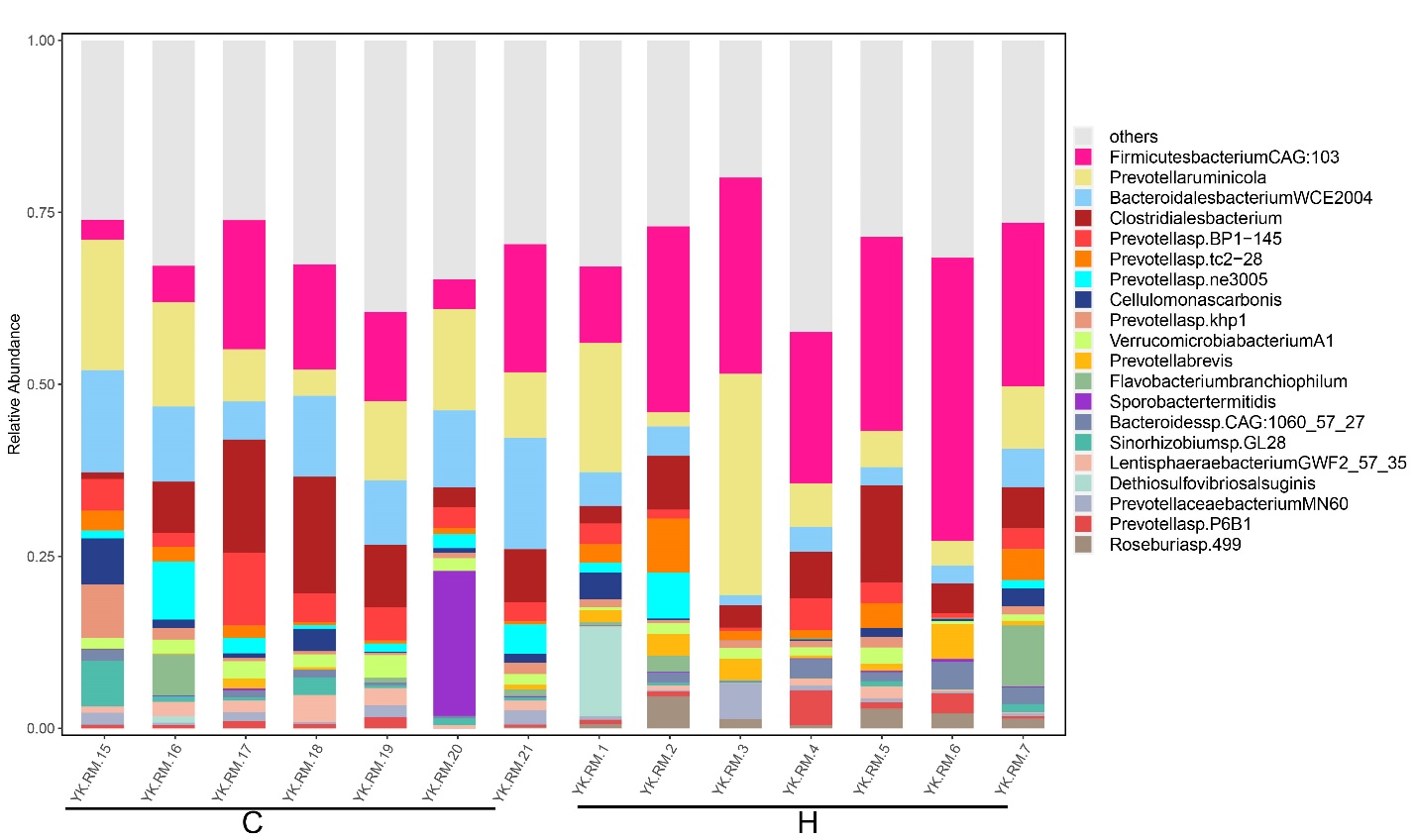

Supplement: Supplementary file 1 [file Table_1.docx]
